# Amidated and Ibuprofen-Conjugated Kyotorphins Promote Neuronal Rescue and Memory Recovery in Cerebral Hypoperfusion Dementia Model

**DOI:** 10.3389/fnagi.2016.00001

**Published:** 2016-01-26

**Authors:** Sónia Sá Santos, Sara M. Santos, Antónia R. T. Pinto, Vasanthakumar G. Ramu, Montserrat Heras, Eduard Bardaji, Isaura Tavares, Miguel A. R. B. Castanho

**Affiliations:** ^1^Instituto de Medicina Molecular, Faculdade de Medicina de LisboaLisboa, Portugal; ^2^Laboratori d’Innovació en Processos i Productes de Síntesi Orgànica, Departament de Química, Universitat de GironaGirona, Spain; ^3^Departamento de Biologia Experimental, Faculdade de Medicina do PortoPorto, Portugal; ^4^Instituto de Biologia Molecular e CelularPorto, Portugal; ^5^i3S – Instituto de Inovação e Investigação em Saúde, Universidade do PortoPorto, Portugal

**Keywords:** 2VO-dementia model, chronic cerebral hypoperfusion, cognitive impairment, hippocampus, kyotorphin derivatives, neuroprotection

## Abstract

Chronic brain ischemia is a prominent risk factor for neurological dysfunction and progression for dementias, including Alzheimer’s disease (AD). In rats, permanent bilateral common carotid artery occlusion (2VO) causes a progressive neurodegeneration in the hippocampus, learning deficits and memory loss as it occurs in AD. Kyotorphin (KTP) is an endogenous antinociceptive dipeptide whose role as neuromodulator/neuroprotector has been suggested. Recently, we designed two analgesic KTP-derivatives, KTP-amide (KTP–NH_2_) and KTP–NH_2_ linked to ibuprofen (IbKTP–NH_2_) to improve KTP brain targeting. This study investigated the effects of KTP-derivatives on cognitive/behavioral functions (motor/spatial memory/nociception) and hippocampal pathology of female rats in chronic cerebral hypoperfusion (2VO-rat model). 2VO-animals were treated with KTP–NH_2_ or IbKTP–NH_2_ for 7 days at weeks 2 and 5 post-surgery. After behavioral testing (week 6), coronal sections of hippocampus were H&E-stained or immunolabeled for the cellular markers GFAP (astrocytes) and NFL (neurons). Our findings show that KTP-derivatives, mainly IbKTP–NH_2_, enhanced cognitive impairment of 2VO-animals and prevented neuronal damage in hippocampal CA1 subfield, suggesting their potential usefulness for the treatment of dementia.

## Introduction

Current estimates indicate that 35.6 million people worldwide are living with dementia, a number that is expected to nearly double every 20 years (World Health Organization [WHO], 2012). Chronic brain ischemia is a prominent risk factor for neurological dysfunction and progression for dementia including Alzheimer’s disease (AD) (Farkas et al., 2007; Institoris et al., 2007; Cechetti et al., 2012). This irreversible disease is characterized by progressive deterioration of cognitive and memory function (Selkoe, 1999; de la Torre, 2004; Blennow et al., 2006). There is accumulating evidence that AD is primarily a vascular disease with neurodegenerative consequences (de la Torre, 2004). The convergence of aging and decreased cerebral perfusion results in a “critically attained threshold of cerebral hypoperfusion” (CATCH), which promotes distortion of brain capillary structure and impairment of NO release (de la Torre and Stefano, 2000). Experimental animal models have been improved to investigate circulation-dependent behavioral deficits resultant of chronic cerebrovascular insufficiency as it occurs in human aging and AD (Farkas et al., 2007). In rats, permanent bilateral common carotid artery occlusion (two-vessel occlusion, 2VO) causes progressive and irreversible cognitive impairment with Alzheimer’s phenotype: learning difficulties, memory loss, failure of neuronal signaling, neuropathological damage in the hippocampus and cerebral cortex within a variable time frame since occlusion (Farkas et al., 2007). In 2VO-model there is neurodegeneration of various cerebral structures, particularly in the CA1 pyramidal cell layer of the hippocampus, a brain region known to be highly implicated in spatial learning and memory (Rolls and Kesner, 2006), and also susceptible to post-ischemic inflammatory phenomena and β-amyloid accumulation (Sekeljic et al., 2012).

Kyotorphin (KTP) is an endogenous dipeptide (L-Tyr-L-Arg) synthesized in nerve terminals, that plays an important role in pain inhibition at the CNS (Takagi et al., 1979a,b; Shiomi et al., 1981; Ueda et al., 1986). To improve KTP delivery to CNS through the manipulation of charge and affinity for lipids (Ribeiro et al., 2012), we have recently succeeded in designing two new KTP-derivatives: KTP-amide (KTP–NH_2_) and IbKTP–NH_2_ (Ribeiro et al., 2011a,b). Both derivatives proved to induce strong analgesic activity following systemic administration (Ribeiro et al., 2011a,b), in contrast with underivatized KTP, without evidences of major side-effects when compared to clinically relevant opioids (Ribeiro et al., 2013). Derivatization seems to enable CNS-targeting. Besides analgesia, it has been hypothesized that KTP has neuromodulating and neuroprotective properties (Nazarenko et al., 1999; Bocheva and Dzambazova-Maximova, 2004), as well as an antiepileptic effect (Godlevsky et al., 1995) and neuroleptic activity affecting calcium-dependent currents in postsynaptic membrane (Santalova et al., 2004).

Due to its L-arginine residue, KTP could also act as substrate for nNOS (NO synthase in neurons), with subsequent formation of NO which would then induce analgesia via met-enkephalin release (Arima et al., 1997). Disruption of NO homeostasis may hasten the development of AD. Actually, prolonged brain hypoperfusion brought on by CATCH seems to promote regional endotheliopathies due to basal deficit of NO, that over time, can evolve to such a degree that lead to AD symptoms and progressive neurodegeneration (de la Torre and Stefano, 2000). Additionally, when neuronal death occurs it may in turn cause a decreased level of endogenous KTP in brain which further impacts on chronic pain and impairment of NO production.

Our recent clinical studies support the existence of a link between AD, pain, and KTP in humans. Indeed, not only we observe that pain is underestimated in AD patients (Santos and Castanho, 2013) but also that KTP has decreased levels in the CSF of AD patients (Santos et al., 2013). Moreover, there was an inverse correlation between levels of phosphorylated-tau protein (biomarker of AD progression) and of KTP (Santos et al., 2013). The present study was conducted to investigate the neuroprotective effects of chronic post-ischemic treatment with KTP–NH_2_ and IbKTP–NH_2_, on motor function, memory impairment, and hippocampal injury in a 2VO-dementia rat model.

## Materials and Methods

### Compounds

Peptides KTP–NH_2_ and IbKTP–NH_2_ were synthesized as described elsewhere (Ribeiro et al., 2011a; Ramu et al., 2014). For animal-surgery, anesthetic, and analgesic drugs were: Imalgene^®^1000 (ketamine 100 mg/ml; Merial, France); Domitor^®^(medetomidine hydrochloride 1 mg/ml; Pfizer, OrionPharma, Finland); Antisedan^®^(atipamezole hydrochloride 5 mg/ml; OrionPharma); Bupaq^®^(buprenorphine 0.3 mg/ml; RichterPharma, Austria).

### Animals and Housing

Young female Sprague–Dawley rats weighing 225–250 g (3-month-old), purchased to Charles River (L’Arbresle Cedex, France), were housed in-group (3–4 per cage) with unrestricted access to water and food, and under controlled conditions (22 ± 2°C; lights on: 7:00 a.m. to 7:00 p.m.). Surgical procedures and behavioral testing were carried out during the light period of the 12 h light–dark cycle.

All described experiments were conducted in compliance with the European Community legislation (Directive 2010/63/EU), and were approved by the Ethical Committee for Animal Research of IMM (Faculty of Medicine, University of Lisbon) and the Portuguese Competent Authority for Animal Welfare (DGAV).

### Surgery: Two-Vessel Carotid Artery Occlusion (2VO) Procedure

One week after animals’ arrival, permanent global ischemia and sham surgery was performed as described elsewhere (Bennett et al., 1998; Vicente et al., 2009). Briefly, animals were anesthetized for surgery with a mixture of ketamine (75 mg/kg BW, i.p.) plus medetomidine (4 mg/kg BW, i.p.). Following ventral midline incision, both common carotid arteries were exposed and carefully separated from their sheats and vagal nerves, and permanently ligated with 5–0 silk sutures. Sham-group was subjected to the same surgical procedures without actual carotid-ligation. After the procedure, rats were injected with the medetomidine-reversing agent mixture (Antisedan^®^; 1 mg/kg BW) and kept on a 37°C heating pad until they recovered from the anesthesia. During the first 24 h post-surgery, buprenorphine was administrated for pain relief purposes (0.05 ml/150–300 g BW q 8–12 h). (More details in Suplemmentary Material).

### Rat Treatment Regimen

KTP–NH_2_ and IbKTP–NH_2_ were dissolved in saline solution (0.9% NaCl, 5% DMSO) prior to i.p. injection (dose volume of 1 ml/kg BW). KTP-derivatives were administrated as a chronic treatment regimen during 7 consecutive days (single i.p. dose/day) in two different timings: (A) 1 week and (B) 4 weeks after the onset of 2VO-surgery. KTP-derivatives were injected only to occluded-animals.

**FIGURE 1:**
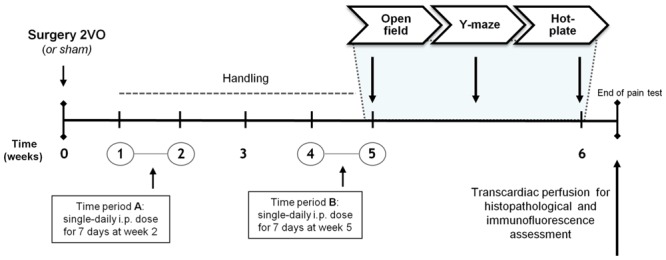
**Time course of the experiments planned.** Female Sprague–Dawley rats were divided into groups according to the type of surgery and to the injected compound during the two time periods A and B: i.e., KTP–NH_2_ (32.3 mg/kg = 96 μmol/kg), IbKTP–NH_2_ (24.2 mg/kg = 46 μmol/kg) or saline solution (vehicle). KTP-derivatives were administrated only to 2VO-animals. Last day of “Time period B” corresponded to the first day of behavioral testing (i.e., open-field test). Abbreviations: i.p., intra-peritoneal; 2VO, two-vessel occlusion.

Animals were matched by body weight and randomly assigned to one of four experimental groups: (1) sham-operated controls (sham group); (2) 2VO-control group; (3) 2VO-animals receiving KTP–NH_2_ i.p. (32.3 mg/kg = 96 μmol/kg); and (4) 2VO-animals receiving IbKTP–NH_2_ i.p. (24.2 mg/kg = 46 μmol/kg). Selected doses of KTP-derivatives were based on our previous results concerning their analgesic action profile (Ribeiro et al., 2011a,b, 2013). Control 2VO- and sham-operated groups were i.p. injected with the vehicle (saline solution). A timeline of experiments is depicted in **Figure 1**.

### Behavioral Test Procedures

At week 6 post-surgery (**Figure 1**), rats were tested in standard behavioral paradigms (in a quiet room with dimmed light). A blind coding was used for the animal groups. (Full details of this section in Supplementary Material).

#### Motor Function Testing: Open-Field

Open-field test (OFT) is a widely used test of locomotion activity, exploratory and anxiety behaviors (Ribeiro et al., 2013). Testing protocol has been described by us in detail previously (Ribeiro et al., 2013). Briefly, 15 min after i.p. injection (with one of KTP-derivatives or with vehicle) rats were placed individually in the center of OFT arena (LWH: 67 cm × 67 cm × 51 cm), behavior was video-recorded for 5 min and motor activity parameters obtained using specific software (Smart version 2.5.10 program; Panlab, Barcelona, Spain). Results are shown as average velocity (i.e., mean velocity with the resting time excluded), % of time resting, number of crossings between two areas inside apparatus and % of time spent in the center of arena (for details see Ribeiro et al., 2013).

#### Memory Testing: Y-Maze

Y-maze is a simple two-trial recognition test for measuring spatial recognition memory skills in rodents (Dellu et al., 2000). Experimental protocol was similar as the one described in (Wang et al., 2006). Briefly, Y-shaped apparatus comprises three identical arms at a 120° angle from each other (arm dimensions, LWH: 35 cm × 10 cm × 20 cm). Those three arms were designated as Start arm, in which the rat starts to explore (always open), Other arm (always open) and Novel arm, which is blocked at the first trial (acquisition phase) but open at the second trial (retention phase). Animal behavior was video-monitored for 5 min (second trial), enabling the number of total entries (sum of entries in all three arms, i.e., Novel+Other+Start) and time spent in Novel and Other arms to be analyzed.

#### Pain Testing: Hot-Plate

Thermal sensitivity evaluation was done using the hot-plate nociception testing. Briefly, immediately after the last i.p. injection animals were placed individually on an 35°C aluminum surface, heated gradually at 9°C/min (cut-off = 52.5°C) (IITC Incremental Hot/Cold plate, Series 8/Software, IITC Life Sciences, San Fernando Valley, CA, USA). The temperature to elicit a hind paw licking or jumping was recorded.

### Histopathology and Immunofluorescence

After the last behavioral test, rats were anesthetized using ketamine/medetomidine mixture and perfused transcardially with 0.9% saline, followed by 4% paraformaldehyde. Brains were removed, post-fixed and coronal 15-μm thickness sections were cut (Leica CM 3050S cryostat, Nussloch, Germany). Sections of dorsal hippocampus (Paxinos and Watson, 2007) were serially collected, with one in every two processed for Hematoxylin-Eosin (H&E) staining or immunofluorescence. The first set were H&E-stained and subsequently observed under a brightfield microscope Leica DM2500 (Wetzlar, Germany) equipped with a digital camera Leica DFC420 for image acquisition (Software Leica FireCam version 3.4.1; 1.25× HCX PL FLUOTAR (NA 0.04) and 5× N PLAN (NA 0.12) dry objectives). Researcher evaluating the histology was blind to the type of treatment the animals had received.

For immunofluorescence studies, the second set of hippocampal sections was double-stained for the astrocytic marker mouse anti-glial fibrillary acidic protein (GFAP, 1:200; Milipore, Temecula, CA, USA) and for the neuronal marker rabbit anti-neurofilament-L protein (NFL, 1:50; Milipore). Secondary antibodies were goat anti-mouse IgG Alexa 488 (1:200; Molecular Probes, Eugene, OR, USA) and goat anti-rabbit IgG Alexa 594 (1:200; Molecular Probes). Sections were counterstained with the DNA stain, Hoechst 33342 (6 μg/ml; Molecular Probes). All samples were analyzed in a Zeiss LSM 510 META confocal point-scanning microscope (Mannheim, Germany) using excitation wavelengths of 405, 488, and 594 nm (for blue, green, and red channels, respectively). Immunofluorescence images were capture (Software LSM 510 version 4.0 SP2; 40× Water Immersion C-Apochromat (NA 1.2) objective) in two randomized areas of dorsal CA1 subfield in both hemispheres, i.e., CA1 images were taken bilaterally in each rat. All acquisition conditions were kept constant between samples during the capture process.

Tissue background was determined and since its autofluorescence was negligible, background subtraction was not required for immunofluorescence quantification.

Maximum intensity Z-stack projection was carried out and ImageJ Fiji 1.48c Software (http://rsb.info.nih.gov) was used to measure the intensities of the fluorescence signals for GFAP (green) and NFL (red), after gray-scale threshold determination. One measurement was taken from each hippocampus of each animal (two measurements per rat, 3–4 rats per group) rendering a total of 6–8 data points per group. The investigator was blind to animals’ experimental condition during image analysis. (Full details in Supplementary Material, including equipment and settings).

### Statistical Analysis

Data are represented as the groups’ mean ± SEM (standard error of the mean).

All statistical analyses were calculated with Prism 6 Software (GraphPad Software, La Jolla, CA, USA). Statistically significant differences were analyzed using two-tailed Student’s *t*-test (unpaired) or one way ANOVA followed by Tukey’s multiple comparison test when indicated. *P* < 0.05 was considered significant.

## Results

### Histopathological and Immunofluorescence Evaluation

**FIGURE 2:**
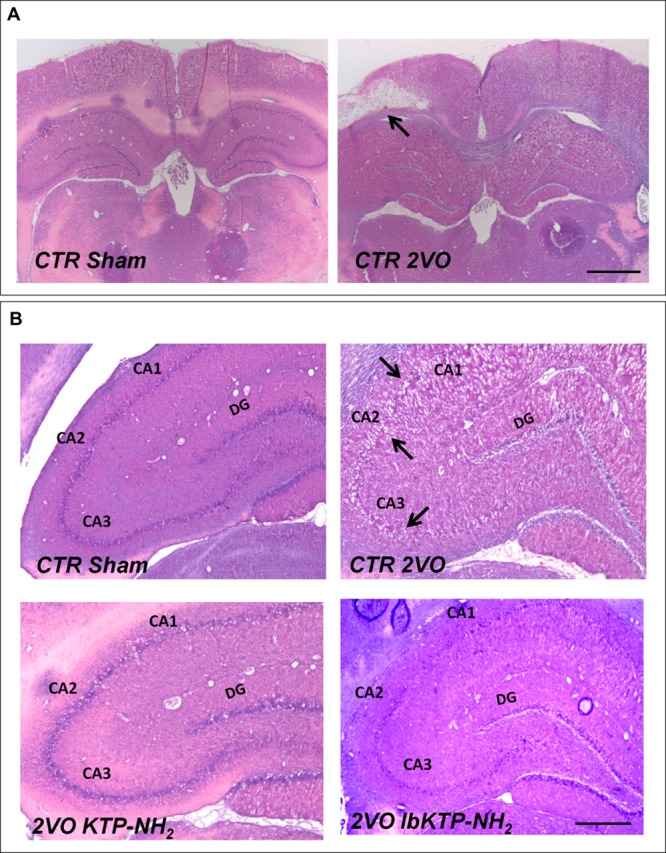
**Representative H&E staining photomicrographs of hippocampal sections from sham-operated and 2VO-female rats treated or not with KTP-derivatives (details in **Figure 1**).** Histological evaluation was performed six weeks after surgery. **(A)**: images showing ischemic lesion (white area, black arrow) in 2VO rat (12.5× magnification). Scale bar: 300 μm. **(B)**: images showing unilateral changes of the CA1, CA2, and CA3 pyramidal cell layers in 2VO-control group (black arrows are pointing at damaged layers) (50× magnification). Scale bar: 80 μm. Abbreviations: CA, cornu ammonis; CTR, control; 2VO, two-vessel occlusion; CTR Sham, control sham-operated animals; DG, dentate gyrus.

Representative photomicrographs of H&E-stained hippocampal sections are shown in **Figure 2**. In some animals subjected to 2VO-surgery, brain tissue loss was observed (**Figure 2A**): ischemic regions were colored white while the non-ischemic regions were colored pink.

2VO-control animals showed significant unilateral changes (right or left hemisphere) in the histoarchitecture of cornu ammonis (i.e., CA1, CA2, and CA3) subfields (**Figure 2B**), namely the loss of pyramidal cells layers. These degenerative changes were not observed in both KTP-treated 2VO groups.

**FIGURE 3:**
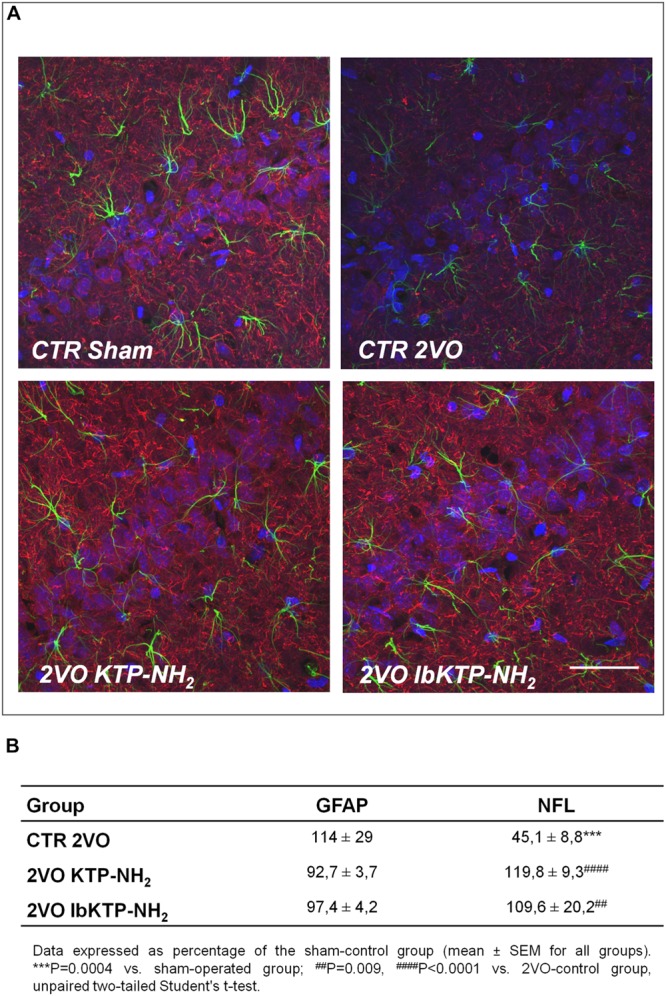
**(A)** Representative confocal Z-stack images (maximum intensity) of imunostaining for GFAP (green, astrocytic marker) and NFL (red, neuronal marker) in the hippocampal CA1 subfield from sham-operated and 2VO-female rats (details in **Figure 1**). Nuclei is stained blue (Hoechst). Upper panels: control groups, sham and 2VO (left and right painel, respectively). Lower painels: KTP-treated 2VO groups, 2VO KTP–NH_2_, and 2VO IbKTP–NH_2_ (left and right painel, respectively) (400× magnification). Note a significant decrease in the NFL signal in the 2VO-control group. Scale bar: 50 μm. **(B)** Quantitative results of GFAP and NFL fluorescence signals. Measurements were performed bilaterally from 3 to 4 rats per group, rendering at least 6 data points per group. Abbreviations: CTR, control; 2VO, two-vessel occlusion; CTR Sham, control sham-operated animals; GFAP, glial fibrillary acidic protein; NFL, neurofilament-L protein.

Immunofluorescence studies were performed to evaluate the effects of KTP-derivatives on astrocytic responses and against neuronal damage in hippocampal CA1 subfield, using as cellular markers, GFAP and NFL, respectively. Representative immunofluorescence images are presented in **Figure 3A**, in which one can observe a lower NFL signal for 2VO-control group compared with other groups. Quantitative analysis of both fluorescence signals (**Figure 3B**) showed no significant effects on GFAP occur in the three 2VO-groups when compared to the sham group. As expected, there was a significant decrease in NFL content in 2VO-control group (**Figure 3B**; *P*-values shown in the table footnotes). In contrast, the NFL immunofluorescence results for both KTP-treated 2VO groups were similar to those in sham group.

### Open-Field Test

**FIGURE 4:**
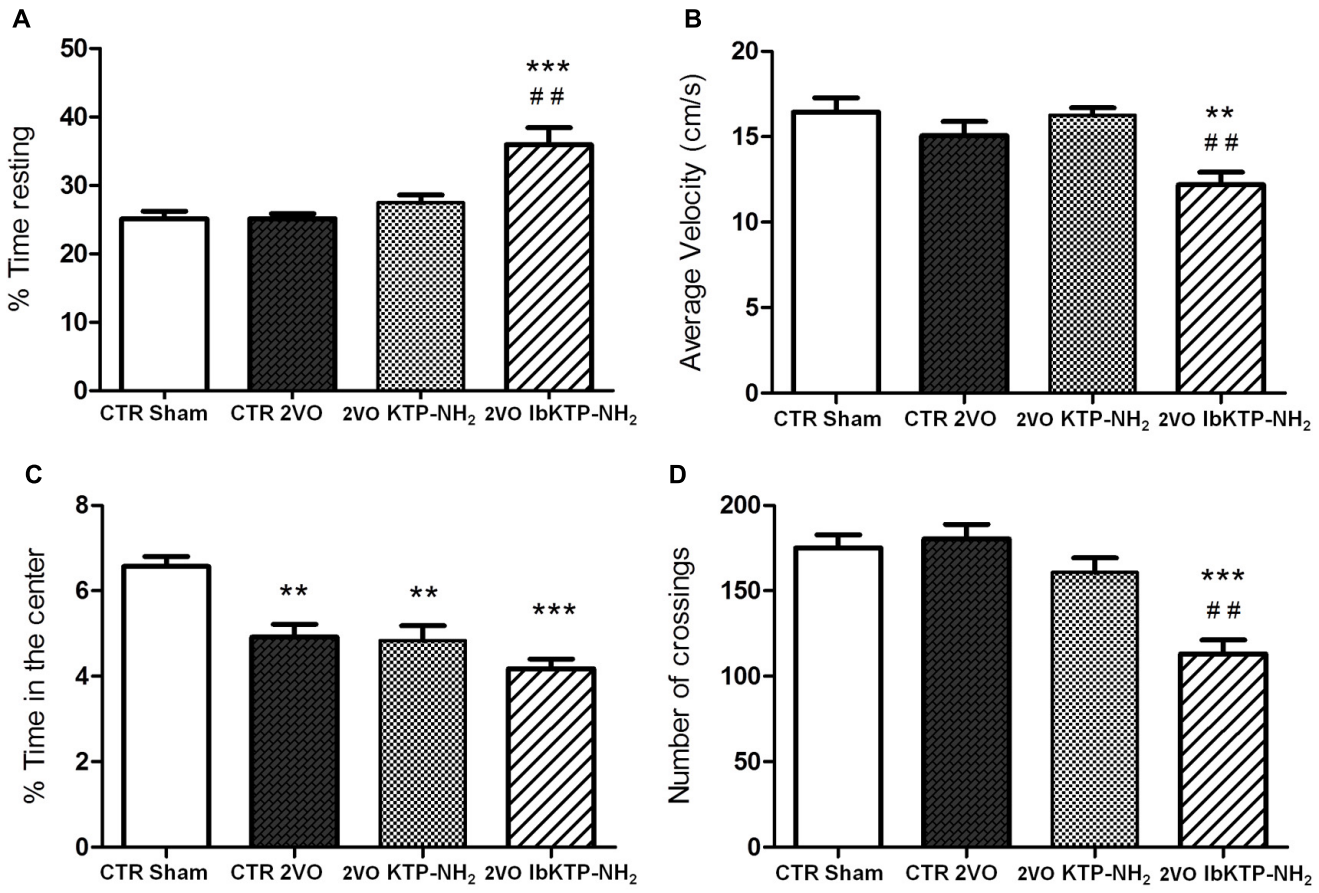
**Locomotion performance of KTP-treated rat females, during the 6th week after the onset of 2VO-surgery (details on chronic treatment regimen in **Figure 1**).** Open-field test (OFT): 2VO-animals were individually placed in the center of the test apparatus 15 min after being i.p. injected with one of KTP-derivatives (KTP–NH_2_ or IbKTP–NH_2_), or vehicle (saline with 5% DMSO used as a control). Sham-operated animals were injected with the vehicle. Behavior was video-recorded for a 5 min time period and data are shown as % time spent resting **(A)**, average velocity **(B)**, % time spent in the center of the arena **(C)**, and the number of crossings **(D)**. In all OFT experiments, *n* = 5 animals per group. In **(A,D)**: ^∗∗∗^*P* < 0.001 vs. 2VO-control and sham-operated groups. In **(B,C)**: ^∗∗^*P* < 0.01, ^∗∗∗^*P* < 0.001 vs. sham-operated group. In **(A,B,D)**: ^##^*P* < 0.01 vs. KTP–NH_2_. Data analyzed using one way ANOVA [*P* = 0.0003 in **(A)**, *P* = 0.0027 in **(B)**, *P* = 0.0001 in **(C,D)**] followed by Tukey’s post test. All data are expressed as mean ± SEM. Abbreviations: CTR, control; 2VO, two-vessel occlusion; CTR sham, control sham-operated animals.

Locomotor activity in a new environment was measured in an open-field apparatus (**Figure 4**). There were no difference between the vehicle-treated animals (i.e., sham-operated and 2VO controls) for the velocity parameter, % of time resting and number crossings. In fact, the only significant difference between the sham-operated rats and the three 2VO-groups was the % of time spent in the center of the arena (**Figure 4C**), i.e., all the 2VO-animals exhibit a more pronounced anxious behavior.

Moreover, the pattern of locomotor response is clearly different in IbKTP–NH_2_-treated 2VO group as those animals moved slower (**Figure 4B**), spent more time resting (**Figure 4A**) and crossed less frequently between areas (**Figure 4D**). We previously reported this effect in normal rats (without any type of surgery, and also after 15 min i.p.) and seems to be due a synergistic effect of ibuprofen and KTP (Ribeiro et al., 2013).

The statistical comparisons, the number of animals and *P*-values are shown in the respective figure legend.

### Y-Maze

**FIGURE 5:**
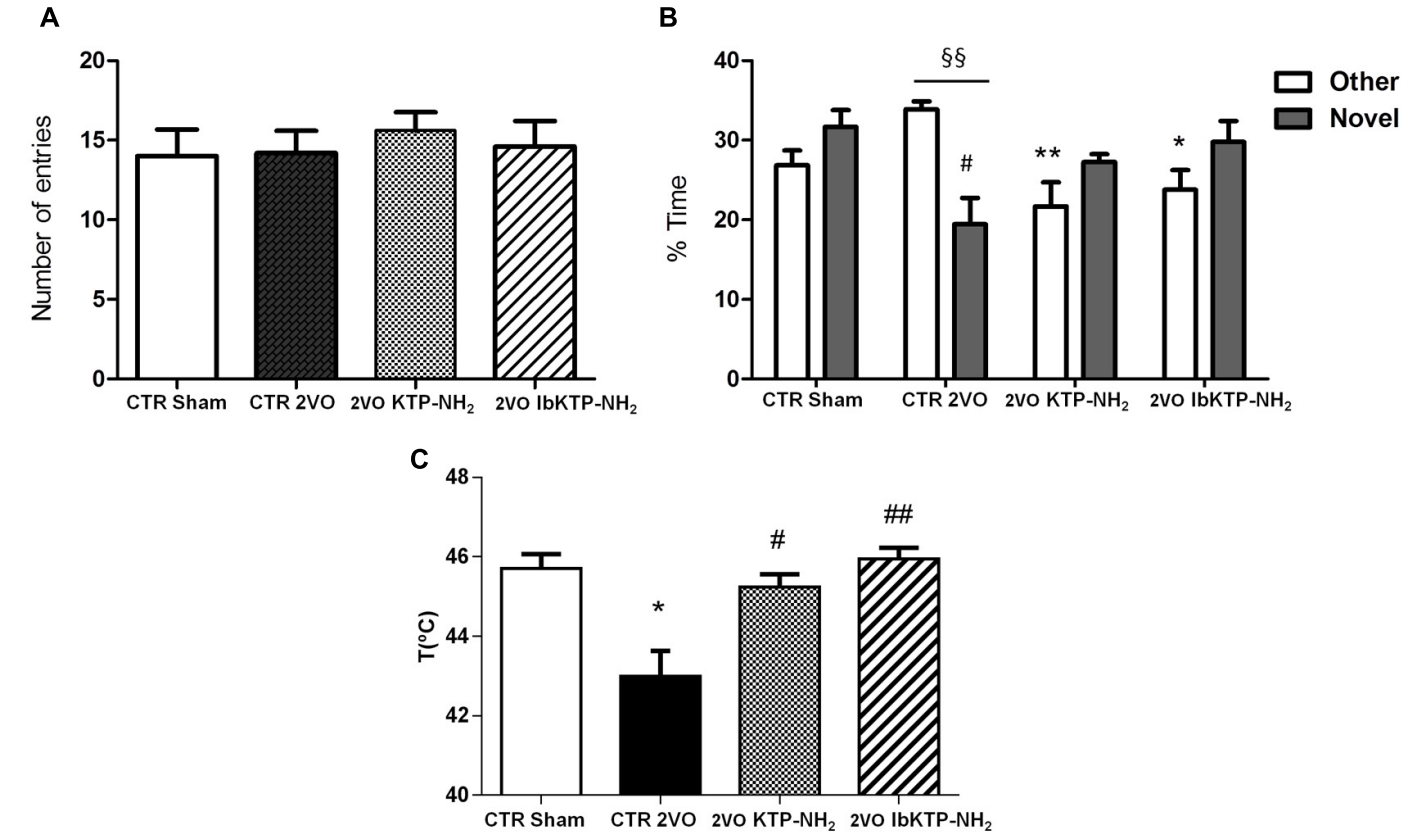
**Cognitive performance in the Y-maze **(A,B)** and pain behavioral responses in the hot-plate **(C)** of KTP-treated rat females during the 6th week after the onset of 2VO-surgery (details on chronic treatment regimen in **Figure 1**). (A,B)** Y-maze: animals were individually placed in the Start arm of the apparatus and allowed to explore freely the entire maze. Behavior was video-monitored for a 5 min time period and data are shown as number of total entries **(A)** and % time spent in the Other and Novel arms **(B)**. In all Y-maze experiments, *n* ≥ 4 animals per group. In **(B)**: ^#^*P* < 0.05 vs. sham-operated group; ^∗^*P* < 0.05, ^∗∗^*P* < 0.01 vs. 2VO-control group, one way ANOVA [*P* = 0.0083 for % time in Other arm, *P* = 0.0283 for % time in Novel arm] followed by Tukey’s post test. Comparison between % time in Other arm and % time in Novel arm for 2VO-control group: ^§§^ *P* = 0.0028, unpaired two-tailed Student’s *t*-test. **(C)** Hot-plate: animals were individually placed in the analgesimeter apparatus and temperature to elicit a hind paw licking or jumping recorded. In all hot-plate experiments, *n* ≥ 3 rats per group ^∗^*P* = 0.0209 vs. sham-operated group; ^#^*P* = 0.0123, ^##^*P* = 0.0026 vs. 2VO-control group, unpaired two-tailed Student’s *t*-test. All data are expressed as mean ± SEM. Abbreviations: CTR, control; 2VO, two-vessel occlusion; CTR sham, control sham-operated animals.

**Figure 5** shows the results of KTP-treated 2VO animals in Y-maze task, in which short-term spatial recognition memory was evaluated.

To determine if the motivation to explore the maze apparatus was the same among the groups during the testing period, the number of total arm entries were analyzed. There was no difference found between all groups in terms of total number of arm entries (**Figure 5A**). Analysis of which arm was chosen to be explored first revealed similar outcomes between sham-operated and IbKTP–NH_2_-treated groups: i.e., all those animals entered the Novel arm as the first choice (100%), whereas the 2VO-control group chose unanimously the Other arm to go first. The percentage of animals that entered Novel arm as first choice in KTP–NH_2_ group was 60%.

Additionally, there was no difference between sham group and KTP-treated 2VO groups for the % of time spent in the Other arm and in the Novel arm (**Figure 5B**). In contrast, the 2VO-control group spent less time in the Novel arm when compared with sham-operated animals. Also, the analysis within each experimental group showed that 2VO-control group explored the Other arm significantly more than Novel arm (**Figure 5B**), whereas all the other groups (sham and KTP-treated 2VO animals) spent similar amount of time both in the Novel and Other arms (**Figure 5B**). The statistical comparisons, the number of animals and *P*-values are shown in the respective figure legend.

### Hot-Plate Test

Sensitivity of sham- and 2VO-groups to painful stimuli was assessed using the hot-plate test. As shown in **Figure 5C**, 2VO-control group exhibited a pronounced sensitivity in hot-plate response, with first signs of pain being observed at lower temperature (43.00 ± 0.64°C), evidenced by the threshold variation decrease of 2–3°C when compared to the other three groups. Similar pain behavior between sham and KTP-treated 2VO groups was observed. The statistical comparisons, the number of animals and *P*-values are in the respective figure legend.

## Discussion

It is now consensual that ischemic episodes in brain trigger a cascade of degenerative events similar to those that ultimately culminate in irreversible dementia of Alzheimer’s phenotype (Pluta et al., 2013). Besides β-amyloid peptide and hyperphosphorylated tau protein accumulation in post-ischemic brains, human epidemiological studies indicated a higher incidence of dementia (i.e., up to ninefold) following a few months of ischemic injury (Pluta et al., 2013).

Permanent bilateral common carotid artery occlusion (2VO) in rats is a suitable experimental model to investigate the neurodegeneration and cognitive consequences of chronic brain hypoperfusion. Like in human aging and dementia, the 2VO-animals show a progressive loss of hippocampal neurons, which in turn leads to cognitive decline and behavioral changes (Farkas et al., 2007). The usefulness of the 2VO-rat model for the development and testing of potentially neuroprotective drugs, against ischemic damage, and/or dementia, has been emphasized (Farkas et al., 2007).

In this work, two analgesic KTP-derivatives, KTP–NH_2_ and IbKTP–NH_2_, were studied regarding their ability in post-ischemia to ameliorate cognitive dysfunction, behavioral functions, and neuronal damage caused by chronic brain hypoperfusion in female rats (2VO-rat model). From a therapeutic standpoint, it is of interest to test their effectiveness after the onset of the ischemic insult. We choose two timepoints during chronic phase of brain hypoperfusion for KTP-derivatives administration (i.e., weeks 2 and 5, **Figure 1**), taking into account the progressive neuropathologic changes in hippocampal CA1/CA3 subfields reported in 2VO-model, i.e., comprising periods of negligible neuronal death and when hippocampal neuronal injury is obvious (Farkas et al., 2007). In addition, the one week treatment window after 2VO-surgery provides a clinically relevant paradigm for ischemic insult/dementia therapy.

The protective effects of KTP-derivatives on hippocampal neurons were shown by H&E staining and NFL immunofluorescence. In 2VO-animals treated with saline solution (2VO-control group) there was a notorious unilateral damage at CA1, CA2, and CA3 subfields reflected by the disappearance of the well-defined layer of pyramidal neurons (**Figure 2**). In contrast, both KTP derivatives were effective in preventing extensive neuronal death at those regions. Immunofluorescence studies were focused on CA1 NFL proteins. Loss of NFL is closely related with the selective vulnerability of CA1 neurons in cerebral ischemia (Nakamura et al., 1992). Futhermore, increased CSF levels of neurofilament proteins (including NFL) has been described as marker of neuronal death and axonal degeneration in several neurological disorders (Scherling et al., 2014). Chronic hypoperfusion caused a significant reduction in the NFL signal in the CA1 subfield (2VO-control group, **Figures 3A,B**). In contrast, no changes were observed for KTP–NH_2_ and IbKTP–NH_2_ groups when compared to the sham-control group.

Cerebral ischemia triggers reactive astrogliosis, a condition characterized by an increase of GFAP levels in astrocytes (Farkas et al., 2007). Although astrocytic activation and proliferation can be detected in the cortex (Farkas et al., 2007) and hippocampus (Cechetti et al., 2012) after 1 week of the occlusion, GFAP increase may be not evident until 6 months later (Farkas et al., 2007). This is in agreement with the absence of major astrogliosis in hippocampal CA1 region after 6 weeks of 2VO-surgery (**Figure 3**).

Although ischemic injury may affect brain areas related to motor function (i.e, cortex and neocortex regions), there are no obvious signs of motor deficits in 2VO-rats (Farkas et al., 2007; Cechetti et al., 2012). Similar results were obtained for control groups (sham-operated and 2VO) in terms of velocity, % of resting and number of crossings, suggesting that bilateral carotid-occlusion did not impair locomotion performance (**Figure 4**). In fact, when tested in the Y-maze arena all the 2VO-groups showed same behavior as the sham-operated ones in the total number of arm visits (another locomotor activity index, (Wang et al., 2006), reinforcing that locomotor function remained intact in 2VO-animals. Nevertheless, all female rats subjected to bilateral occlusion were more anxious than the sham-controls in the open-field, as measured by decreased time spent in the center of the arena. These results are in agreement with previous studies of global ischemia, in which tested animals developed an anxious behavior (Dhooper et al., 1997). Clinical studies have shown mood alterations and increased anxiety levels in AD patients (Teri et al., 1999). Hippocampus plays a crucial role in many species-typical behaviors (like anxiety), potentially influencing performance in a variety of behavioral tests (Castelhano et al., 2013). Indeed, hippocampal damage disrupts spatial cognition and may induce increased motor activity in rodents. Previous studies indicate that even small lesions in dorsal CA1 subfield of mice lead to dramatic spatial memory impairments in the Y-maze and hyperactivity upon exposure to a novel environment (Dillon et al., 2008). Herein, the anxious behavior of 2VO-operated animals was not affected by the administration of KTP-derived compounds, showing that these peptides probably do not possess intrinsic anxiolytic properties, and/or did not act on key areas responsible for this behavior.

Spatial recognition memory of female rats was measured by the two-trial Y-maze test (Dellu et al., 2000; Wang et al., 2006). Y-maze paradigm is based on an innate tendency of rodents to explore a novel environment but not on learning a new behavior or rule, allowing to measure behavior parameters such as recognition vs. discrimination memory and spatial exploration (Dellu et al., 2000; Wang et al., 2006). Rodents typically prefer to investigate a new arm of the maze (unfamiliar) rather than returning to one that was previously visited (familiar). Therefore, if memory and novelty-seeking behavior are not affected, animals are expected to enter the Novel arm more than the Other arm. In our study, although all animals shown to be equally motivated to explore the Y-maze (total entries into all arms were similar), their response to novelty inside the apparatus was not the same. A significant difference was detected between groups related to the first arm choice and percent of time spent in Novel vs. Other arms with the 2VO-control group spending more time and choosing the Other arm to go first (**Figure 5B**). This indicates that 2VO-surgery induced short-term memory deficits, making those females unable to discriminate novelty and familiarity. Conversely, both KTP-treated groups and sham-animals chosen more often first the Novel arm and spent similar time in the Novel vs. Other arm. In fact, the percentage of animals that entered the Novel arm as the first choice was 100% in both sham and IbKTP–NH_2_ groups and 60% in KTP–NH_2_ group. Since the first choice for Novel arm reflects recognition of the unfamiliar arm (discrimination memory), is obvious that administration over time of IbKTP–NH_2_ and KTP–NH_2_ improved the ability of 2VO-animals to distinguish the Novel arm from the familiar ones. Therefore, IbKTP–NH_2_ and KTP–NH_2_ treatment enhanced spatial recognition memory in 2VO animals, with a greater effect of IbKTP–NH_2_.

A direct correlation between cerebral hypoperfusion-induced memory deficits and hippocampal CA1 neuronal damage has been reported (Bennett et al., 1998; De Jong et al., 1999; Cechetti et al., 2012; Xi et al., 2014). In addition, Y-maze memory performance in female rats (same strain as the one used herein) is more tightly coupled with CA1 morphology than CA3 subfield when compared to male rats (McLaughlin et al., 2005). Taken all together, one concludes: improved memory abilities seen in KTP-derivatives-treated 2VO female rats is due to the neuroprotective effects of peptides on CA1 neurons.

Several evidences support that hippocampus processes nociception-related information, including the affective-emotional component involved (McKenna and Melzack, 2001). Dorsal hippocampal CA1 neurons respond to persistent noxious stimuli, and specific neuronal-receptor antagonists microinjections into the dentate gyrus and CA1 region produce analgesia (McKenna and Melzack, 2001; Soleimannejad et al., 2006). Therefore, it is likely that alterations in hippocampal structure/function, such as the ones induced by 2VO-surgery, lead to changes in pain perception. Herein, the hot-plate test (acute pain model) was used to assess nocifensive response to thermal stimuli. Our data shows a similar nociception outcome between sham-operated and KTP-treated 2VO groups, whereas 2VO-vehicle group displayed significant higher sensitivity in thermal response (**Figure 5C**). At first, the latter findings seem to contrast with evidences of increasead hot-plate latencies in rats with frontal/cingulate cortex lesions (i.e., disruption of supraspinally integrated responses) (Pastoriza et al., 1996). However, we can not exclude, despite efforts to habituate all animals to the analgesimeter, the lower pain threshold in 2VO-vehicle females may reflect an anxiety-related behavior (an aggravating factor for experimental/clinical pain) (Le Bars et al., 2001).

## Conclusion

Our experimental findings clearly show that KTP-derivatives improved cognitive impairment and prevented neuronal damage in hippocampal CA1 subfield induced by chronic cerebral hypoperfusion. Moreover, IbKTP–NH_2_ showed to be more effective in restoring normal cognitive function than KTP–NH_2_. The mechanism underlying this repair and/or recovery seems to involve, in addition to improved permeability across lipid bilayers (Serrano et al., 2015) and other factors, the presence of the ibuprofen in IbKTP–NH_2_ derivative, which may attenuate some of neuroinflammatory processes in 2VO-ischemic brain. Epidemiological studies have shown that long-term NSAIDs-based therapies reduce the risk of developing AD (Gasparini et al., 2004). Also, several NSAIDs including ibuprofen protect neurons against mitochondrial Ca^2+^ overload induced by β-amyloid oligomers (Yu et al., 2009). In rodent AD models, chronic administration of ibuprofen prevents oxidative damage, significantly inhibits amyloid formation and deposition, and improves cognitive functions (Gasparini et al., 2004; Wilkinson et al., 2012). Further studies are needed to unveil which are the molecular targets of IbKTP–NH_2_.

## Author Contributions

Study design: SSS, SMS, MC. Data collection and analysis: SSS, SMS, AP. Data interpretation: SSS, SMS, IT, MC. Paper drafting: SSS, SMS, IT, MC. Paper revising: SSS, SMS, MH, EB, IT, MC. Synthesis and purification of KTP-derivatives: VR, MH, EB. Final approval of the version to be published: SSS, SMS, AP, VR, MH, EB, IT, MC. Agreement to be accountable for all aspects of the work in ensuring that questions related to the accuracy or integrity of any part of the work are appropriately investigated and resolved: SSS, SMS, AP, VR, MH, EB, IT, MC.

## Conflict of Interest Statement

The authors declare that the research was conducted in the absence of any commercial or financial relationships that could be construed as a potential conflict of interest.

## Abbreviations

AD: Alzheimer’s disease
BBB: blood–brain barrier
CA: cornu ammonis
CATCH: critically attained threshold of cerebral hypoperfusion
CNS: central nervous system
CSF: cerebrospinal fluid
DMSO: dimethyl sulfoxide
GFAP: glial fibrillary acidic protein
H&E: hematoxylin and eosin
IbKTP–NH_2_: KTP–NH_2_ linked to Ibuprofen
i.p.: intraperitoneal
KTP: kyotorphin
KTP–NH_2_: kyotorphin-amide
LWH: length, width, height
NFL: neurofilament-L (low–molecular weight) protein
NO: nitric oxide
nNOS: nitric oxide synthase located in neurons
OFT: open-field test
2VO: permanent bilateral common carotid artery occlusion, two-vessel occlusion
