# Supplementary material for: Amidated and Ibuprofen-Conjugated Kyotorphins Promote Neuronal Rescue and Memory Recovery in Cerebral Hypoperfusion Dementia Model
Source: Front Aging Neurosci. 2016 Jan 26;8:1. doi: 10.3389/fnagi.2016.00001 (PMC4726799; doi:10.3389/fnagi.2016.00001)
Supplement: Supplementary file 1 [file Data_Sheet_1.DOCX]

Supplementary Material

**Amidated and ibuprofen-conjugated kyotorphins promote neuronal rescue and memory recovery in cerebral hypoperfusion dementia model**

**Sónia Sá Santos, Sara M. Santos, Antónia R.T. Pinto, Vasanthakumar G. Ramu, Montserrat Heras, Eduard Bardaji, Isaura Tavares and Miguel A.R.B. Castanho***

*** Correspondence:** Miguel A.R.B. Castanho: macastanho@medicina.ulisboa.pt

# Supplementary Data: Materials and methods

**Surgery: two-vessel carotid artery occlusion (2VO) procedure:** During the 2VO- or sham-surgery, the animals were breathing spontaneously and body temperature maintained around 37°C by a regulating heating pad. Afterwards, all animals were returned to their home cages with free access to food and water. Animals were closely monitored during the post-operative recovery for appearance, activity, feeding behavior and body weight. Like in larger surgical interventions, 2VO is typically followed by an initial decrease in body weight (Farkas et al., 2007) but all animals involved in this study regained their weight before behavioral testing, which was performed five weeks after 2VO procedure. Body weight was measured before the surgery and controlled whenever the animals were handled for i.p. injection and/or experimentation.

**Behavioral test procedures:** Behavioral studies were carried out between 9 a.m. and 6 p.m. in animals accustomed to the testing room and to researchers performing the trials. All behavioral apparatus were thoroughly cleaned after each rat to mitigate olfactory stimuli. The experimenter was never present in the room while the animals explored the open-field and the maze. At the day of experiments, animals were brought into the testing room for at least 2 h prior to the start of the behavioral session. Researchers conducting and/or evaluating the behavioral tests were blind to the group assignment of the experimental animals.

***Motor function testing: Open-field***. Open-field apparatus consisted of an empty square box (LWH: 67x67x51 cm), “virtually” divided in three concentric squares: borders (near the walls), periphery and center. To evaluate the number of crossings, the open field arena was also virtually divided into 16 equal rectangles, and the number of times each animal crossed between two areas was measured. Each animal was considered to be resting if the mean velocity was < 3 cm/s. All animals were tested only once.

***Memory testing: Y-Maze.*** The Y-maze test consisted of two trials: the first one had 10 min duration and allowed the animal to explore freely only two arms (Start and Other) with Novel arm blocked with black Plexiglas. Then, after an interval of 1 h, the rat was placed in same starting arm, with free access to all three arms for 5 min (2nd trial). Animal bedding was used to cover the floor of the maze and was mixed after each individual trial to prevent rats from using odor cues in maze navigation. Entry was considered to be complete when all four limbs were within an arm. No animal had jumped out of the maze arena during the testing period.

**Histopathology and Immunofluorescence:** Following transcardiac perfusion, animals were decapitated and their brains were carefully removed, maintained for post-fixation in the same fixative solution at 4°C for 24 h and then cryoprotected with a 30% sucrose solution for at least two days. Brains were gelatin-embedded and then sectioned at a thickness of 15-µm on a cryostat (Leica CM 3050S). Only the coronal sections located at the level of dorsal hippocampus (around - 3.6-mm posterior from bregma (Paxinos and Watson, 2007) were collected, mounted on SuperFrost® Plus slides (Menzel-Glaser, Braunschweig, Germany). H&E staining was performed in the first set of sections, in order to detect the location of areas of ischemic brain damage, as well as to examine changes in laminar structures of the hippocampus namely pyramidal cell layers in the cornu ammonis (i.e., CA1, CA2 and CA3) subfields. For the immunofluorescence studies in the second set of sections, first samples were placed in PBS for 10 min at 37^o^C to remove gelatin from brain tissue. Sections were subsequently treated with 0.1% Triton X-100 in PBS (PBS-Tx) for membrane permeabilization, blocked for 1 h with 2% bovine serum albumin (BSA) in PBS at room temperature (RT), and then incubated at 4^0^C overnight with the mixture of primary antibodies GFAP (1:200; catalog #MAB3402, Milipore, Temecula, CA, USA) plus NFL (1:50; catalog #AB9568, Milipore) prepared in blocking solution (2% BSA in PBS). In the following day, sections were washed in PBS-Tx and incubated with the secondary antibodies goat anti-mouse IgG Alexa 488 (1:200; catalog #A11017, Molecular Probes, Eugene, OR, USA) and goat anti-rabbit IgG Alexa 594 (1:200; catalog #A11012, Molecular Probes) for 1 h at RT in a humified dark chamber. For nuclei staining, sections were incubated with Hoechst 33342 (6µg/ml; catalog #H1399; Molecular Probes) for 10 min at RT protected from light. At the end, sections were cover slipped with ProLong® Gold antifade reagent (Molecular Probes). Negative controls in which the primary antibodies were omitted were performed simultaneously.

**Additional information about equipment and settings:**

**Figure 2 (Histology) -** Leica DM2500 microscope with a digital camera Leica DFC420 (Software Leica FireCam version 3.4.1; 1.25x [HCX PL FLUOTAR](http://www.e-leica.com/Objectives.nsf/showObjectivePage?openagent&id=4BB5910D53F74DA8C1257507004FAC8A) (NA 0.04) and 5x [N PLAN](http://www.e-leica.com/Objectives.nsf/showObjectivePage?openagent&id=8572F44993CF050FC1257507004FADBE) (NA 0.12) dry objectives; 2592 x 1944 resolution; bit depth 24).

**Figure 3A (Immunofluorescence) -** Zeiss LSM 510 META confocal point-scanning microscope (Software LSM 510 version 4.0 SP2; 40x Water Immersion C-Apochromat (NA 1.2) objective**;** maximum intensity Z-stack projection: Z-step size 0.57-µm; Z slices range: 24 to 32 slices; 1024 x 1024 resolution; bit depth 8).

Fluorochromes: excitation wavelengths of 405 (blue), 488 (green) and 594 (red) nm. Dichroic beamsplitters: blue channel HFT 405/488/561 (emission filter BP 420-480); green channel HFT 405/488/561 - secondary dichroic NFT 490 (emission filter BP 505-530); red channel HFT 405/488/594 - secondary dichroic NFT 565 (emission filter LP 615).

**Supplementary Material References:**

Farkas, E., Luiten, P. G., and Bari, F. (2007). Permanent, bilateral common carotid artery occlusion in the rat: a model for chronic cerebral hypoperfusion-related neurodegenerative diseases. *Brain Res. Rev.* 54, 162-180.

Paxinos, G., and Watson, C. (2007). *The Rat Brain in Stereotaxic Coordinates*. Sixth ed., San Diego: Elsevier.
